# Indications to total thyroidectomy for multinodular goiter in old patients

**DOI:** 10.1186/1471-2318-11-S1-A49

**Published:** 2011-08-24

**Authors:** A Puzziello, AM Lucisano, R Gervasi, C Siani, MA Lerose, G Orlando, N Innaro, G Vescioe, R Sacco

**Affiliations:** 1 UU.OO di Chirurgia Generale e di Endocrinochirurgia, Università Magna Grecia di Catanzaro, Italy

## Background

In Western society, the percentage of elderly people is continually growing. The prevalence of goiter increases with the age and it is estimated that 90% of women over 60 years old and 60% of men over 80 years old have a relief of thyroid nodules. This has great importance for these patients, because the incidence of malignant transformation is higher than younger ones and these are often tumor very aggressive patterns. If thyroidectomy is indicated for patients with suspected neoplasm and severe obstructive symptoms, their surgery should not be delayed since a late urgent operation could raise morbidity and mortality risk. The main indications for young patients are due to obstructive and metabolic causes over and above suspected cancer.

Total thyroidectomy is considered by many authors as the treatment of choice.

## Materials and methods

75 elderly patients were submitted to thyroidectomy. The indications were metabolic (42.6%), obstructive (32%) and for suspected cancer (25.4%).

## Results

The most frequent complications observed with respect to young patients in different series have been cardiovascular, pulmonary or urological. Regarding the complications directly related to thyroidectomy, there were no differences compared to younger groups, except transient complications (hypoparathyroidism, seroma). In our experience, the main complication was represented by hypocalcemia (30.6%), permanent in 8% of cases. Cancer was relieved in 21.3% of cases.

Prognosis has been excellent in most cases, with immediate remission of symptoms related to thyrotoxicosis and to tracheal and esophageal compression in almost all symptomatic patients.

## Conclusions

Age is an independent prognostic factor for cancers. It has been demonstrated that elderly patients with PTC that are operated have better prognosis and quality of life due to the resolution of dyspnea and dysphagia. In our experience, we think that age is not a contraindication to thyroid surgery.
